# *In vitro* Bactericidal Activities of Combination Antibiotic Therapies Against Carbapenem-Resistant *Klebsiella pneumoniae* With Different Carbapenemases and Sequence Types

**DOI:** 10.3389/fmicb.2021.779988

**Published:** 2021-12-13

**Authors:** Jocelyn Qi-Min Teo, Nazira Fauzi, Jayden Jun-Yuan Ho, Si Hui Tan, Shannon Jing-Yi Lee, Tze Peng Lim, Yiying Cai, Hong Yi Chang, Nurhayati Mohamed Yusoff, James Heng-Chiak Sim, Thuan Tong Tan, Rick Twee-Hee Ong, Andrea Lay-Hoon Kwa

**Affiliations:** ^1^Department of Pharmacy, Singapore General Hospital, Singapore, Singapore; ^2^Saw Swee Hock School of Public Health, National University of Singapore and National University Health System, Singapore, Singapore; ^3^Singhealth Duke-NUS Pathology Academic Clinical Programme, Singapore, Singapore; ^4^Singhealth Duke-NUS Medicine Academic Clinical Programme, Singapore, Singapore; ^5^Department of Microbiology, Singapore General Hospital, Singapore, Singapore; ^6^Department of Infectious Diseases, Singapore General Hospital, Singapore, Singapore; ^7^Emerging Infectious Diseases, Duke-National University of Singapore Medical School, Singapore, Singapore

**Keywords:** *in vitro*, bactericidal, combination, carbapenemase, enterobacterales, tigecycline, polymyxin

## Abstract

Carbapenem-resistant *Klebsiella pneumoniae* (CRKP) is becoming increasingly problematic due to the limited effectiveness of new antimicrobials or other factors such as treatment cost. Thus, combination therapy remains a suitable treatment option. We aimed to evaluate the *in vitro* bactericidal activity of various antibiotic combinations against CRKP with different carbapenemase genotypes and sequence types (STs). Thirty-seven CRKP with various STs and carbapenemases were exposed to 11 antibiotic combinations (polymyxin B or tigecycline in combination with β-lactams including aztreonam, cefepime, piperacillin/tazobactam, doripenem, meropenem, and polymyxin B with tigecycline) in static time-kill studies (TKS) using clinically achievable concentrations. Out of the 407 isolate-combination pairs, only 146 (35.8%) were bactericidal (≥3 log_10_CFU/mL decrease from initial inoculum). Polymyxin B in combination with doripenem, meropenem, or cefepime was the most active, each demonstrating bactericidal activity in 27, 24, and 24 out of 37 isolates, respectively. Tigecycline in combination with β-lactams was rarely bactericidal. Aside from the lower frequency of bactericidal activity in the dual-carbapenemase producers, there was no apparent difference in combination activity among the strains with other carbapenemase types. In addition, bactericidal combinations were varied even in strains with similar STs, carbapenemases, and other genomic characteristics. Our findings demonstrate that the bactericidal activity of antibiotic combinations is highly strain-specific likely owing to the complex interplay of carbapenem-resistance mechanisms, i.e., carbapenemase genotype alone cannot predict *in vitro* bactericidal activity. The availability of WGS information can help rationalize the activity of certain combinations. Further studies should explore the use of genomic markers with phenotypic information to predict combination activity.

## Introduction

*Klebsiella pneumoniae* are common Gram-negative pathogens that are implicated in a variety of infections including pneumonia, bloodstream infections, and skin/soft tissue infections. As one of the ESKAPE organisms, it possesses the ability to acquire multiple resistance mechanisms to the various drug classes and is a major contributor to nosocomial infections (Rice, 2010). Resistance to carbapenems, one of the last-line antimicrobial agents, in these bacteria has resulted in very limited treatment options for these infections. Although there are currently a few novel agents such as ceftazidime/avibactam and meropenem/vaborbactam, they are not universally active against all carbapenem-resistant *K. pneumoniae* (CRKP) and are cost-prohibitive or are not readily available in certain settings (Bush and Bradford, 2019). Treatment with antibiotic combinations is regarded as the optimal alternative, especially in patients with high mortality risks (Giannella et al., 2019). Multiple *in vitro* studies evaluating combination therapy in CRKP infections have been conducted with varying results (Lenhard et al., 2016). Previously, we have shown that antibiotic combinations were highly strain-specific in extensively drug-resistant NDM-producing *K. pneumoniae* (Lim et al., 2015), while *in vitro* synergy of double carbapenem combinations have been widely demonstrated, albeit primarily in KPC-producing *K. pneumoniae* (Bulik and Nicolau, 2011; Chua et al., 2015; Oliva et al., 2016).

The management of CRKP infections is complicated by the various mechanisms mediating carbapenem resistance which include: (1) production of carbapenemases (e.g., KPC, metallo-β-lactamases, OXA-48); (2) extended-spectrum β-lactamases (ESBLs) in combination with mutations that alter porin function or expression; and (3) overexpression of efflux pumps (Papp-Wallace et al., 2011). Even among the carbapenemases, there are differences in the types of substrates, the mechanisms of hydrolysis, and the hydrolytic activities of the active substrates (Queenan et al., 2010; Jeon et al., 2015). For instance, OXA carbapenemases have a weaker activity against carbapenems compared to the other carbapenemases (Queenan et al., 2010). Along with the type of antibiotics selected for combination therapy, this carbapenemase diversity may have implications in the efficacy of antibiotic combination therapy (Poirel et al., 2016). Hence, this study sought to evaluate the *in vitro* activity of various antibiotic combinations against CRKP with different carbapenemase genotypes.

## Materials and Methods

### Bacterial Isolates

Thirty-seven CRKP isolates with varied carbapenemases were tested. The majority of these isolates were selected from an ongoing carbapenem resistance surveillance project conducted at a 1,800-bed public healthcare hospital since 2015. The remaining isolates were received at the hospital’s pharmacy research laboratory for antibiotic combination testing, including isolates from various other local hospitals (Cai B. et al., 2016). These isolates were representative of difficult-to-treat infections encountered which will likely require combination therapy. They possessed highly resistant phenotypic profiles [carbapenem minimum inhibitory concentrations (MICs) ≥ 8 mg/L] and represented various high-risk international clones (e.g., ST11, ST17, ST14, ST20, ST147, and ST231) with varying carbapenemases.

Genus identity was determined at the hospital’s microbiology laboratory as part of routine investigations using VITEK GNI+ cards (bioMérieux, Hazelwood, MO, United States). The isolates were stored at −70°C in Microbank™ (Pro Lab Diagnostics Inc., Ontario, Canada) storage vials and sub-cultured twice on 5% blood agar plates (Thermo Fisher Scientific Microbiology, Malaysia) for 24 h at 35°C prior to each experiment.

This study is exempted from review by the Singhealth Centralized Institutional Review Board, as it is a retrospective study involving archival bacterial isolates, which does not fall under the Human Biomedical Research Act. No identifiable data were collected.

### Antibiotics

Aztreonam, meropenem, and polymyxin B were purchased from Toronto Research Chemicals. Cefepime was purchased from Kemimac(s) Pte Ltd. Piperacillin/tazobactam and tigecycline were purchased from Sigma-Aldrich. Doripenem was obtained from Shionogi and Co. Aliquots of stock solutions of all antibiotics were prepared in sterile water and stored at −80°C. Before each experiment, the aliquots were thawed and diluted to the desired concentrations with cation-adjusted Mueller Hinton broth (Ca-MHB).

### *In vitro* Susceptibility Testing

Carbapenem non-susceptibility was detected routinely at the microbiology laboratory using either disk diffusion testing or the VITEK ^®^ 2 instrument. The minimum inhibitory concentrations (MICs) were determined in this study using customized commercial microbroth dilution panels (Trek Diagnostics, East Grinstead, United Kingdom). *E. coli* ATCC 25922 was used as the quality control strain. MICs were interpreted according to the Clinical and Laboratory Standards Institute (CLSI) guidelines, except for tigecycline which was interpreted according to the Food and Drug Administration (FDA) criteria for tigecycline (CLSI, 2020).

### Molecular Characterization

CRKPs were routinely tested for the presence of carbapenemase genes either at the hospital’s microbiological laboratory or at the National Public Health Laboratory using in-house polymerase chain reaction (PCR)-based assays or the Cepheid Xpert ^®^ Carba-R assay on the GeneXpert ^®^ device (Cepheid, Sunnyvale, CA, United States).

Genomic DNA was extracted from overnight bacterial cultures and purified with the Qiagen Blood DNeasy kit (Qiagen Inc., Valencia, CA, United States). The genomic DNA was then used to prepare libraries for paired-end whole-genome sequencing using the Illumina HiSeq or MiSeq instrument (Illumina, San Diego, CA), with a resultant sequencing depth of at least 50-fold. Sequence types (STs) were determined by performing a basic local alignment search tool (BLAST) search of the assembled contigs against multilocus sequence typing (MLST) databases^1^, while other antimicrobial resistance features were characterized using the Kleborate tool (v.2.0.4)^2^.

### Static Time-Kill Studies

Modified TKS were performed on the isolates with the antibiotics singly and in two-antibiotic combinations using procedures described previously (Cai Y. et al., 2016; Cai et al., 2017) to examine the bactericidal activity. In brief, log-phase bacterial suspensions were diluted into 15 mL of fresh Ca-MHB to yield an initial inoculum of approximately 5 log_10_CFU/mL, which were then transferred to flat-bottomed sterile flasks containing 1 mL of antibiotic solutions and placed into a shaker water bath at 35°C.

A total of 11 combinations were tested—polymyxin B or tigecycline was tested in combination with five different β-lactams. Polymyxin B was also tested in combination with tigecycline. The concentrations utilized in this study were derived from clinically relevant unbound concentrations when maximum antibiotic doses were administered (Supplementary Table 1).

At 24 h, aliquots were obtained in duplicates from each flask. Total viable counts were enumerated visually by plating serial dilutions of the aliquots on Mueller-Hinton agar plates (Thermo Fisher Scientific, Singapore). The final limit of detection was 1.3 log_10_CFU/mL. Bactericidal activity was defined as a 3 log_10_ CFU/mL decrease (99.9% kill) in the colony count from the initial inoculum at 24 h (CLSI, 1999).

## Results

### Isolate Characteristics

The phenotypic characteristics of the 37 isolates are presented in Table 1. All isolates had similar β-lactam phenotypic characteristics where there was phenotypic resistance to all β-lactams tested. The minimum inhibitory concentrations (MICs) to aztreonam, cefepime, and piperacillin-tazobactam were uniformly high (≥64 mg/L). Carbapenem MICs were also high in all isolates (8 to ≥ 32 mg/L, MIC_50_: ≥ 32 mg/L). Polymyxin B and tigecycline resistance were observed in nine (24.3%) and four (10.8%) isolates, respectively, of which one isolate was resistant to both polymyxin B and tigecycline (EC301).

**TABLE 1:** Phenotypic characteristics (antibiotic susceptibilities) of 37 CRKP.

Strain	Carbapenemase	Minimum inhibitory concentrations (mg/L)			
		Doripenem	Meropenem	Polymyxin B	Tigecycline
EC1642	None	8	16	**≥16**	2
EC0283	None	16	16	2	2
EC0215	OXA-181	≥32	≥32	0.5	2
EC1717	OXA-181	≥32	≥32	0.5	2
EC2096	OXA-181	16	≥32	**8**	≤0.25
EC1277	OXA-181	16	≥32	0.5	0.5
EC1824	OXA-181	≥32	≥32	0.5	1
EC1812	OXA-181	≥32	≥32	0.5	1
EC0633	OXA-232	≥32	≥32	1	2
EC1902	OXA-232	≥32	≥32	**≥16**	1
EC0307	KPC-2	8	≥32	0.5	1
EC0301	KPC-2	≥32	≥32	**≥16**	**≥16**
EC2772	KPC-2	16	≥32	≤0.25	≤0.25
EC1470	KPC-2	≥32	≥32	**≥16**	2
EC2617	KPC-2	≥32	≥32	0.5	**8**
EC0174	NDM-1	≥32	≥32	0.5	2
EC0044	NDM-1	≥32	≥32	≤0.25	0.5
EC0466	NDM-1	≥32	≥32	2	**4**
EC0045	NDM-1	≥32	≥32	0.5	2
EC0177	NDM-1	≥32	≥32	0.5	2
EC0178	NDM-1	≥32	≥32	0.5	2
EC0334	NDM-1	≥32	≥32	2	0.5
EC1170	NDM-1	≥32	≥32	**8**	2
EC0172	NDM-1	≥32	≥32	0.5	≤0.25
EC0299	IMP-1	≥32	≥32	1	**4**
EC0360	NDM-1 + OXA-181	≥32	≥32	0.5	2
EC0564	NDM-1 + OXA-181	≥32	≥32	**8**	1
EC0567	NDM-1 + OXA-181	≥32	≥32	**8**	1
EC0391	NDM-1 + OXA-181	≥32	≥32	**8**	1
EC1488	NDM-1 + OXA-232	≥32	≥32	2	2
EC1522	NDM-1 + OXA-232	≥32	≥32	0.5	1
EC1645	NDM-1 + OXA-232	≥32	≥32	0.5	2
EC1655	NDM-1 + OXA-232	≥32	≥32	0.5	1
EC1678	NDM-1 + OXA-232	≥32	≥32	0.5	1
EC1729	NDM-1 + OXA-232	≥32	≥32	1	1
EC1792	NDM-1 + OXA-232	≥32	≥32	0.5	1
EC0462	NDM-1 + OXA-232	≥32	≥32	0.5	1

*Aztreonam and piperacillin-tazobactam minimum inhibitory concentrations are not shown here as all isolates have values ≥ 64 mg/L (resistant phenotype). Values in bold denote polymyxin- and/or tigecycline-resistant isolates.*

**FIGURE 1:**
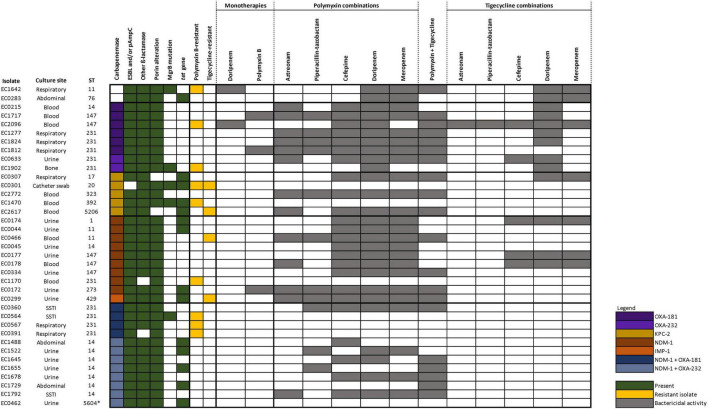
Genotypic characteristics and bactericidal activities of the various antibiotic regimens against 37 CRKP. Only doripenem and polymyxin B monotherapies are displayed in the figure as all other monotherapy regimens did not demonstrate bactericidal kill.

The genotypic characteristics are summarized in Figure 1 (genotypic details of individual isolates are presented in Supplementary Table 2). A total of 14 different sequence types (STs) were included. All except two isolates were carbapenemase-producing. Among the various CRKP with differing carbapenemase genotypes, the majority harbored an extended-spectrum β-lactamase (ESBL), most commonly CTX-M-15, together with porin alteration. Out of the nine polymyxin-resistant isolates, MgrB mutations were detected in five of them. Tetracycline resistance *tet* genes were observed in 14 isolates which included both tigecycline-susceptible and -resistant isolates. None of the isolates harbored plasmid-mediated resistance genes associated with polymyxin (*mcr*) and tigecycline [*tet*(X)] resistance.

### Static Time-Kill Studies Results

The activity of each antibiotic alone was limited against most of the strains except in two isolates (EC1642 and EC2096) where doripenem (corresponding to a high dose extended infusion regimen) resulted in a bactericidal kill; and in three isolates (EC1717, EC1812, and EC0172) where polymyxin B resulted in bactericidal kill (Figure 1 and Supplementary Table 3). Consequently, doripenem- or polymyxin-containing combination regimens exhibited bactericidal killing against these isolates, respectively.

Of the 407 combinations evaluated, only 146 (35.9%) exhibited bactericidal killing at 24 h. Polymyxin with doripenem (27/37 isolates), meropenem (24/37 isolates), cefepime (24/37 isolates), and tigecycline (20/37 isolates) were the combinations exhibiting the highest bactericidal activities. Polymyxin B in combinations with the various β-lactams were more active (99/185 bactericidal activity, 53.5%) than tigecycline combinations (27/185 bactericidal activity, 14.6%), while polymyxin and tigecycline demonstrated bactericidal activities in 20/37 (54.0%) isolates.

Against polymyxin- and/or tigecycline-resistant isolates, only 32/121 (26.4%) combinations were bactericidal, while 114/286 (39.9%) combinations were bactericidal against isolates that remained susceptible to both polymyxin B and tigecycline. This indicates that combinations were less likely to be active in resistant isolates, suggesting that polymyxin or tigecycline resistance phenotypes could be predictive of the activity of polymyxin and tigecycline combinations, respectively. Only seven polymyxin B combinations retained bactericidal activity against polymyxin-resistant isolates (Polymyxin B + doripenem against EC1642, EC2096, EC1902; polymyxin B + meropenem against EC1642; polymyxin B + cefepime against EC2096 polymyxin B + tigecycline against EC1642, EC2096). Against tigecycline-resistant isolates, polymyxin + tigecycline was the only tigecycline-containing combination that exhibited bactericidal killing (EC2617 and EC0299).

Analyzing only the polymyxin B- and tigecycline-susceptible isolates where monotherapy was not bactericidal (22 isolates), our results did not reveal marked differences in bactericidal activity between isolates harboring OXA-48-like, KPC-2 or NDM-1 (Supplementary Figure 1). Polymyxin B with cefepime, doripenem, or meropenem was bactericidal against almost all of these isolates (except EC0283 where polymyxin + cefepime was not bactericidal). The remaining combinations were variable in activity. Against NDM and OXA dual producers, all combinations were variable in activity. This was despite dual carbapenemase-producing isolates belonging to the same ST and harboring similar genotypic characteristics (carbapenemases, β-lactamases, and porin genes), suggesting that STs were unlikely to predict any specific antibiotics in combination.

Interestingly, only four combinations (polymyxin with meropenem/doripenem or tigecycline with meropenem/doripenem) were bactericidal against EC0283, which did not harbor any carbapenemase. In this study, we included CRKP (carbapenem MICs > 8 mg/L), which were isolates where most single antibiotic therapies including high-dose carbapenem extended infusions will likely fail; hence it is likely EC0283, while not a carbapenemase-producer, harbored higher levels of CTX-M-15 and a higher degree of porin loss to manifest the high carbapenem phenotypic resistance which could not be overcome by combination therapies.

## Discussion

CRKP infections are challenging to treat due to limited treatment options. Antibiotic combination therapy has been explored as a viable option in several *in vitro* studies, but available data are limited by the overrepresentation of KPC producers (Zusman et al., 2013). It is well known that the effectiveness of antibiotic combinations is not universal and tends to be unpredictable, rendering it extremely challenging to select an antibiotic combination regimen. Interactions observed when antibiotics are combined can range from antagonism to synergism rates up to 80% (Zusman et al., 2013; Lenhard et al., 2016; Mohammadi et al., 2017; Jiang et al., 2018). These interactions can be influenced by pathogen factors (species, susceptibility, and resistance mechanisms), antibiotic factors (number, classes, and concentration), and the testing methodology (Zusman et al., 2013). As the understanding of the mechanisms behind the bactericidal/synergistic/additive effect of combinations remains poor, we evaluated 37 CRKP isolates with differing carbapenemase genotypes against 11 two-antibiotic combinations in this study.

In our study, bactericidal activity was observed with at least one polymyxin-containing combination for the majority of the isolates. This result corroborates other *in vitro* studies where synergistic/bactericidal activity has been demonstrated with polymyxin B-containing combinations. Synergy rates between 30 and 59% for *K. pneumoniae* have been reported, and polymyxins in combination with carbapenems have demonstrated bactericidal activity in several *in vitro* studies (Zusman et al., 2013; Lenhard et al., 2016; Scudeller et al., 2021). The utility of polymyxin combinations has mechanistic plausibility. In Gram-negative bacteria like *K. pneumoniae*, most antibiotics enter the cell *via* porin channels in the outer membrane. Polymyxins’ main mechanism of bacterial killing has been suggested to be the disruption/destabilization of the outer membrane (Trimble et al., 2016). There is evidence that synergism between polymyxin and other antibiotics occurs as a result of this membrane disruption, allowing the entry of the partner antibiotics into the bacterial cell (Rosenthal and Storm, 1977). However, it appears that bactericidal activity of polymyxin combinations is primarily limited to polymyxin-susceptible isolates in our study, unlike other reports which established combination activity in polymyxin-resistant strains (Jernigan et al., 2012). The difference in combination activities observed in our isolates with frank polymyxin B resistance (mediated by MgrB mutations) might be related to differences in the mechanisms mediating polymyxin resistance.

In contrast, we did not observe good results with tigecycline-containing combinations, even in tigecycline-susceptible isolates. Previous studies have also demonstrated variable *in vitro* tigecycline activity (Pournaras et al., 2011). Antagonism has also been reported with tigecycline and meropenem/doripenem combinations (Bi et al., 2019). Tigecycline is a bacteriostatic drug that exerts its activity *via* ribosomal binding, leading to the prevention of protein synthesis and retardation of cell growth (Greer, 2006). Studies have demonstrated that tetracyclines affect cell division, leading to growth stasis forming the basis of antagonism when paired with bactericidal drugs such as β-lactams which are the most potent against actively dividing cells (Ocampo et al., 2014). This phenomenon, also known as phenotypic tolerance (Tuomanen, 1986), might explain the lack of activity in the tigecycline and β-lactam combinations assessed here, whereas tigecycline when paired with polymyxin still exhibit moderate activity (Supplementary Table 3). Contrary to our expectation of beta-lactam activity being inhibited/antagonized by the addition of tigecycline in tigecycline and beta-lactam combinations, we have also noticed higher 24-h bacterial counts of tigecycline combinations compared to tigecycline monotherapy. The mechanisms behind this antagonism warrant further exploration.

There was high variability in bactericidal activity of the various combinations in our isolates, emphasizing the high strain specificity of antibiotic combinations. It was suggested that genotypic information could be more predictive of combination antibiotic activities/interactions than the phenotypes alone (Shields et al., 2015; Wistrand-Yuen et al., 2020). Knowledge of the carbapenemase family can aid in the rationalization of therapeutic choices since the different carbapenemases have different substrate activities (Queenan and Bush, 2007; Livermore et al., 2020). In this study, aside from the poor bactericidal activity observed amongst the co-producers, we were unable to identify a clear trend among the isolates with the other carbapenemase types, indicating that the knowledge of carbapenemase types alone was a poor indicator of combination activity in our isolates.

The mechanisms of carbapenem resistance are complex and multi-factorial. Aside from being mediated by carbapenemase production, resistance may also result from various combinations of β-lactamases production, porin loss/downregulation, and efflux activity, leading to the same carbapenem resistance phenotype (Codjoe and Donkor, 2017). The availability of WGS results in this study shed some light on our observations. All of our isolates harbored at least one ESBL/plasmid AmpC, in addition to the carbapenemases which might have explained the higher frequency of bactericidal activity in doripenem, meropenem, and cefepime combinations since these β-lactams are generally more stable against ESBL production. We also noted that many of our isolates have porin mutations which may lead to decreased porin expression. The variability in combination effectiveness may be related to differentiation in the levels of porin expression, which was unfortunately not quantitated in this study. Given that the mechanism of combination antibiotic synergism/bactericidal effect is likely due to the increased effective entry of the antibiotic into the bacterial cell, combination therapy may likely be more effective in strains where phenotypic resistance is contributed to a larger extent by cell permeability which may then be “reversed” with antibiotic combinations. In light of this, further studies characterizing/quantitating porin expression and efflux activity may be useful to establish if genetic mechanisms related to cell permeability may be a better predictor of the bactericidal activity of the combination.

The complexity of mechanisms mediating carbapenem resistance has contributed to the difficulty in antibiotic combination selection. However, it is unlikely that there is a universal combination that is effective against all or even the majority of the CRKP strains, and knowledge of the genomic characteristics still only serves as a small step toward the rational selection of antibiotic combinations. Given that our local isolates tend to co-harbor ESBLs and are porin-deficient, partner antibiotics that are ESBL-stable and have better cell penetration profiles should be selected. In our study, polymyxin and doripenem appear to be the most reliable combination against the various types of CRKP. Aside from the better β-lactamase stability of doripenem compared to the other β-lactams like cefepime, aztreonam, and piperacillin-tazobactam, its pharmacodynamic and safety profile of doripenem has allowed the drug to be given at a high-dose prolonged infusion (concentration of doripenem used in this study corresponded to a 2 g every 8-hourly dosing regimen given as a 4-h prolonged infusion), which will likely result in a higher probability in achieving a longer *f*T > MIC (Strawbridge and Nailor, 2016). Furthermore, doripenem MICs tended to be one to twofold lower compared to meropenem. It was also proposed that there might be improved *in vivo* efficacy compared to the other carbapenems due to a favorable immunological profile (enhanced neutrophil killing and reduced endotoxin release) (Hilliard et al., 2011). When taken together with other studies supporting the positive interactions with polymyxin-doripenem combinations (Deris et al., 2012; Jernigan et al., 2012; Lee and Burgess, 2013), this combination may be considered a rational choice for the treatment of CRKP infections, especially if other potentially active agents such as ceftazidime-avibactam are not available. Furthermore, this was the only combination that potentially exhibited activity against polymyxin-resistant strains.

This study is not without limitations. We utilized static time-kill studies to evaluate bactericidal activity, which may not correlate well with *in vivo* studies. The small sample size also limits the generalization of our results to the larger CRKP population. Ideally, further studies, including pharmacokinetic/pharmacodynamic models, animal models, and even clinical trials, should be conducted to verify if these *in vitro* observations may be translated to clinical utility. Nevertheless, the findings are in line and lend support to several other *in vitro* studies as discussed above. We hope the results here may serve as a proof of concept and provide a preliminary guide for rational antibiotic combination design, aiding to narrow down the potential combinations that will eventually be brought to large clinical trials.

## Conclusion

The present study confirmed the high strain specificity of antibiotic combinations among CRKP with various carbapenemase genotypes. Bactericidal killing was observed with polymyxin combinations, in particular, polymyxin B with doripenem, against CRKP with varying carbapenemase genotypes. However, bactericidal killing was rare against polymyxin-resistant CRKP and those harboring more than one carbapenemase, suggesting that more efforts need to be directed at identifying therapeutic options for this group of pathogens. WGS provided genomic information about the bacterial resistome, which when taken together with pharmacokinetic/pharmacodynamic knowledge of the various antibiotics can guide the rational selection of combination antibiotic therapy. This approach will improve the chances of selecting a successful combination through identifying potential synergistic mechanisms and avoidance of antagonism. Future *in vitro* pharmacokinetic/pharmacodynamic studies should incorporate genomic characterization to facilitate comparisons between studies.

## Data Availability Statement

The datasets presented in this study can be found in online repositories. The names of the repository/repositories and accession number(s) can be found below: https://www.ncbi.nlm.nih.gov/, PRJNA577535.

## Author Contributions

JT conceived and designed the study, performed the laboratory work and data curation and data and bioinformatics analyses, and wrote the manuscript. NF, JH, and ST undertook the laboratory work and data curation. SL performed the molecular experiments. HC and NMY performed the time-kill studies and phenotypic characterization. TL and YC designed the experiments and reviewed the manuscript. JS provided the isolates, contributed to the implementation of the research, and reviewed the manuscript. TT reviewed the manuscript. RO supervised the bioinformatics analyses and reviewed the manuscript. AK conceived the study, reviewed the manuscript, and provided funding. All authors read and approved the final manuscript.

## Conflict of Interest

AK received unrestricted funding for research from Pfizer Inc., and Merck Sharp and Dohme (I.A) Corp for investigator-initiated research studies which are not related to this study. The funders were not involved in the study design, collection, analysis, interpretation of data, the writing of this article or the decision to submit it for publication. The remaining authors declare that the research was conducted in the absence of any commercial or financial relationships that could be construed as a potential conflict of interest.

## Publisher’s Note

All claims expressed in this article are solely those of the authors and do not necessarily represent those of their affiliated organizations, or those of the publisher, the editors and the reviewers. Any product that may be evaluated in this article, or claim that may be made by its manufacturer, is not guaranteed or endorsed by the publisher.

## Footnotes

1
https://pubmlst.org/databases/

2
https://github.com/katholt/Kleborate
