# Supplementary material for: In vitro Bactericidal Activities of Combination Antibiotic Therapies Against Carbapenem-Resistant Klebsiella pneumoniae With Different Carbapenemases and Sequence Types
Source: Front Microbiol. 2021 Dec 13;12:779988. doi: 10.3389/fmicb.2021.779988 (PMC8713045; doi:10.3389/fmicb.2021.779988)
Supplement: Supplementary file 1 [file Data_Sheet_1.docx]

**Supplementary materials**

| **Table S1.** Antibiotic concentrations used in time-kill studies | | | |
| --- | --- | --- | --- |
| Antibiotic | Stimulated dosing regimen | Concentration (mg/L) | Reference |
| Aztreonam | 8 g every 24 h (infused over 24 h) | 24 | (1) |
| Cefepime | 2 g every 8 h | 50 | (2) |
| Piperacillin-tazobactam | 4.5 g every 6 h (infused over 4 h) | 35/7 | (3) |
| Doripenem | 2 g every 8 h (infused over 4 h) | 26 | (4) |
| Meropenem | 2 g every 8 h (infused over 3 h) | 20 | (5) |
| Polymyxin B | 30,000 IU/kg/day or at least 1 MU every 12 h | 2 | (6) |
| Tigeycline | 100 mg every 12 h | 2 | (7) |

| **Table S2.** Relevant genomic characteristics of 37 CRKP isolates | | | | | | | | | | | | | |
| --- | --- | --- | --- | --- | --- | --- | --- | --- | --- | --- | --- | --- | --- |
| Strain | Culture site | ST | Carbapenemase | Extended-spectrum 𝛽-lactamase | | | Other 𝛽-lactamase | | | Porin alteration | | MgrB | Tet |
|  |  |  |  | CTX-M | AmpC | SHV | OXA | SHV | TEM | OmpK35 | OmpK36 |  |  |
| EC1642 | Respiratory | 11 | None | CTX-M-15 | DHA-1 |  | OXA-1 | SHV |  |  | Y | Y |  |
| EC0283 | Abdominal | 76 | None | CTX-M-15 |  |  | OXA-1 |  |  | Y | Y |  | Tet(A) |
| EC0215 | Blood | 14 | OXA-181 | CTX-M-15 | CMY-4 |  | OXA-1; OXA-9 | SHV |  | Y | Y |  |  |
| EC1717 | Blood | 147 | OXA-181 | CTX-M-15 |  |  |  | SHV | TEM-1 | Y | Y |  |  |
| EC2096 | Blood | 147 | OXA-181 | CTX-M-15 |  |  |  | SHV |  | Y | Y |  |  |
| EC1277 | Respiratory | 231 | OXA-181 | CTX-M-15 | CMY-4 |  |  | SHV |  | Y | Y |  |  |
| EC1824 | Respiratory | 231 | OXA-181 | CTX-M-15 | CMY-4 |  |  | SHV | TEM-1 | Y | Y |  |  |
| EC1812 | Respiratory | 231 | OXA-181 | CTX-M-15 | CMY-4 |  |  | SHV | TEM-1 | Y | Y |  |  |
| EC0633 | Urine | 231 | OXA-232 | CTX-M-15 |  |  |  |  | TEM-1 | Y | Y |  |  |
| EC1902 | Bone | 231 | OXA-232 |  |  | SHV-12 |  |  | TEM-1 | Y | Y | Y |  |
| EC0307 | Respiratory | 17 | KPC-2 | CTX-M-15 |  |  | OXA-1 |  |  |  |  |  | Tet(A) |
| EC0301 | Swab | 20 | KPC-2 |  |  |  | OXA-1 | SHV |  | Y | Y | Y | Tet(A) |
| EC2772 | Blood | 323 | KPC-2 | CTX-M-15 |  |  | OXA-1 | SHV |  |  | Y |  |  |
| EC1470 | Blood | 392 | KPC-2 | CTX-M-15 |  |  | OXA-1 | SHV |  | Y | Y | Y | Tet(A) |
| EC2617 | Blood | 5206 | KPC-2 | CTX-M-15 |  |  | OXA-1 | SHV |  |  |  |  | Tet(A) |
| EC0174 | Urine | 1 | NDM-1 | CTX-M-14/15 |  | SHV-12 |  |  | TEM-1 | Y |  |  | Tet(A); Tet(G) |
| EC0044 | Urine | 11 | NDM-1 | CTX-M-15 |  |  | OXA-1; OXA-9 | SHV | TEM-1 | Y |  |  | Tet(A) |
| EC0466 | Blood | 11 | NDM-1 | CTX-M-15 |  |  | OXA-1 | SHV | TEM-1 | Y |  |  |  |
| EC0045 | Urine | 14 | NDM-1 | CTX-M-15 |  |  | OXA-1; OXA-9 |  | TEM-1 | Y | Y |  |  |
| EC0177 | Urine | 147 | NDM-1 | CTX-M-15 |  |  | OXA-1 | SHV | TEM-1 | Y |  |  |  |
| EC0178 | Blood | 147 | NDM-1 | CTX-M-15 |  |  | OXA-1 | SHV | TEM-1 | Y |  |  |  |
| EC0334 | Urine | 147 | NDM-1 | CTX-M-15 |  |  | OXA-1; OXA-9 | SHV | TEM-1 | Y |  |  |  |
| EC1170 | Blood | 231 | NDM-1 | CTX-M-15 |  |  |  |  |  | Y | Y |  |  |
| EC0172 | Urine | 273 | NDM-1 | CTX-M-15 |  |  | OXA-1 | SHV | TEM-1 | Y |  |  | Tet(A) |
| EC0299 | Urine | 429 | IMP-1 | CTX-M-15 |  |  | OXA-1 | SHV |  |  | Y |  | Tet(A) |
| EC0360 | SSTI | 231 | NDM-1; OXA-181 | CTX-M-15 | CMY-4 |  |  |  | TEM-1 | Y | Y |  |  |
| EC0564 | SSTI | 231 | NDM-1; OXA-181 | CTX-M-15 | CMY-4 |  |  | SHV | TEM-1 | Y | Y | Y |  |
| EC0567 | Respiratory | 231 | NDM-1; OXA-181 | CTX-M-15 | CMY-4 | SHV-12 |  |  | TEM-1 | Y | Y |  |  |
| EC0391 | Respiratory | 231 | NDM-1; OXA-181 | CTX-M-15 | CMY-4 | SHV-12 |  |  |  | Y | Y |  |  |
| EC1488 | Abdominal | 14 | NDM-1; OXA-232 | CTX-M-15 |  |  | OXA-1; OXA-9 | SHV | TEM-1 | Y | Y |  | Tet(D) |
| EC1522 | Urine | 14 | NDM-1; OXA-232 | CTX-M-15 |  |  | OXA-1; OXA-9 | SHV | TEM-1 | Y | Y |  | Tet(D) |
| EC1645 | Urine | 14 | NDM-1; OXA-232 | CTX-M-15 |  |  | OXA-1; OXA-9 | SHV | TEM-1 | Y | Y |  |  |
| EC1655 | Urine | 14 | NDM-1; OXA-232 | CTX-M-15 |  |  | OXA-1; OXA-9 | SHV | TEM-1 | Y | Y |  | Tet(D) |
| EC1678 | Urine | 14 | NDM-1; OXA-232 | CTX-M-15 |  |  | OXA-1; OXA-9 | SHV | TEM-1 | Y | Y |  |  |
| EC1729 | Abdominal | 14 | NDM-1; OXA-232 | CTX-M-15 |  |  | OXA-1; OXA-9 | SHV | TEM-1 | Y | Y |  | Tet(D) |
| EC1792 | SSTI | 14 | NDM-1; OXA-232 | CTX-M-15 |  |  | OXA-1 | SHV | TEM-1 |  | Y |  |  |
| EC0462 | Urine | 5604 | NDM-1; OXA-232 | CTX-M-15 |  |  | OXA-1 | SHV | TEM-1 |  | Y |  | Tet(D) |
| Y - indicates presence of mutation | | | | | | | | | | | | | |

| **Table S3.** 24 hr Time-kill results | | | | | | | | | | | | | | | | | | | |
| --- | --- | --- | --- | --- | --- | --- | --- | --- | --- | --- | --- | --- | --- | --- | --- | --- | --- | --- | --- |
| Strain | Bactericidal concentration (log10 CFU/mL) | | | | | | | | | | | | | | | | | | |
|  | 0h | Δ24 hr | | | | | | | | | | | | | | | | | |
|  |  | Monotherapy | | | | | | | PMB combinations | | | | | | TGC combinations | | | | |
|  |  | ATM | TAZ | FEP | DOR | MEM | PMB | TGC | ATM | TAZ | FEP | DOR | MEM | TGC | ATM | TAZ | FEP | DOR | MEM |
| EC1642 | 5.38 | 3.62 | 3.62 | 3.62 | -5.38 | 2.60 | 3.62 | 3.62 | 3.62 | 3.62 | 3.62 | **-3.84** | -5.38 | -3.40 | 3.62 | 1.45 | 1.20 | **-4.73** | -4.73 |
| EC0283 | 5.12 | 3.88 | 3.88 | 3.88 | -0.45 | -0.33 | -1.97 | 3.88 | **1.45** | -0.09 | -2.39 | **-3.56** | -5.12 | **3.88** | 3.88 | **3.88** | 3.88 | -5.12 | **-3.91** |
| EC0215 | 5.02 | 4.35 | 3.26 | 4.35 | 2.76 | 3.97 | 2.00 | 3.72 | -5.02 | -2.06 | -5.02 | -5.02 | -5.02 | 0.13 | 4.27 | **4.11** | 3.98 | -3.72 | -0.53 |
| EC1717 | 5.20 | 3.80 | 3.80 | 3.80 | 2.80 | 3.80 | -5.20 | -1.01 | **-5.20** | **-5.20** | **-5.20** | **-5.20** | **-5.20** | **-5.20** | 0.02 | **2.80** | -2.64 | -5.20 | -1.55 |
| EC2096 | 5.34 | 3.66 | 3.66 | 3.66 | -5.34 | 3.66 | 3.66 | -1.84 | 3.66 | 3.66 | -3.28 | **-4.25** | 3.66 | -4.03 | -4.17 | -5.34 | -5.34 | **-5.34** | -5.34 |
| EC1277 | 5.32 | 3.68 | 3.68 | 3.68 | 1.06 | 1.68 | 2.83 | -0.71 | -5.32 | -5.32 | -5.32 | -5.32 | -5.32 | -5.32 | -0.88 | -1.87 | -0.83 | -3.09 | -1.65 |
| EC1824 | 5.23 | 3.77 | 3.77 | 3.77 | -1.64 | 3.77 | -0.43 | -1.06 | -5.23 | -5.23 | -5.23 | -5.23 | -5.23 | -5.23 | -1.56 | **3.77** | -1.52 | -3.78 | 0.82 |
| EC1812 | 5.10 | 3.90 | 3.90 | 3.90 | 2.08 | 3.90 | -5.10 | -0.84 | **-5.10** | **-5.10** | **-5.10** | **-5.10** | **-5.10** | **-5.10** | -1.03 | -0.17 | 0.25 | -2.46 | -0.40 |
| EC0633 | 5.32 | 3.68 | 3.68 | 3.68 | 0.72 | 3.54 | 1.52 | 3.68 | -5.32 | 2.65 | -5.32 | -5.32 | -4.13 | -5.32 | -0.87 | 3.68 | -4.32 | -5.32 | 2.62 |
| EC1902 | 5.29 | 3.97 | 3.92 | 3.79 | 2.47 | 2.96 | 2.97 | -0.61 | 3.71 | 3.71 | -1.32 | -5.29 | 2.71 | -2.20 | **3.71** | **3.71** | -1.72 | -3.29 | -1.73 |
| EC0307 | 5.25 | 3.62 | 3.62 | 3.62 | -2.87 | 0.43 | 3.32 | 3.62 | 3.75 | 3.46 | -5.25 | -5.25 | -3.32 | -5.25 | 3.75 | 3.75 | -2.93 | -5.25 | -3.65 |
| EC0301 | 5.07 | 3.93 | 3.93 | 3.93 | 3.97 | 4.23 | 4.14 | 3.93 | 3.93 | 3.93 | -1.98 | 3.98 | 4.06 | 0.61 | 3.93 | 3.93 | 3.93 | 3.93 | 3.93 |
| EC2772 | 5.46 | 3.55 | 3.55 | 3.59 | 3.60 | 3.73 | -0.29 | -1.00 | -5.46 | -3.29 | -5.46 | -4.66 | -3.82 | -5.46 | -0.63 | -0.77 | -0.58 | -2.04 | -0.92 |
| EC1470 | 5.62 | 3.38 | 3.38 | 3.38 | 3.38 | 3.38 | 3.38 | 3.38 | 3.38 | 3.38 | 3.38 | 2.38 | 3.38 | 2.38 | 2.38 | 3.38 | 3.38 | 2.38 | 3.38 |
| EC2617 | 5.40 | 3.60 | 3.60 | 3.60 | 3.27 | 3.35 | -0.23 | 3.60 | -3.36 | -0.95 | -5.40 | -5.40 | -4.09 | -5.40 | 2.60 | 3.60 | 2.60 | 1.30 | 2.03 |
| EC0174 | 5.13 | 3.87 | 3.87 | 3.87 | 3.87 | 3.87 | -1.69 | 2.77 | -0.09 | -0.90 | -5.13 | -5.13 | -5.13 | -0.98 | 4.24 | 2.55 | -5.13 | -5.13 | -5.13 |
| EC0044 | 5.07 | 4.19 | 4.24 | 4.17 | 3.96 | 3.59 | 2.74 | -1.16 | 0.69 | -0.99 | -5.07 | -5.07 | -5.07 | -0.58 | **4.11** | 0.06 | -2.50 | -0.88 | -1.05 |
| EC0466 | 5.22 | 3.78 | 3.78 | 3.78 | 3.77 | 3.71 | -1.16 | 3.78 | -5.22 | -4.57 | -5.22 | -5.22 | -5.22 | -5.22 | 3.78 | 3.78 | 3.78 | 3.78 | 3.78 |
| EC0045 | 5.19 | 3.81 | 3.81 | 3.81 | 3.81 | 3.81 | -0.94 | 4.19 | 0.15 | -0.54 | -5.19 | -5.19 | -5.19 | -2.36 | 4.14 | 2.91 | -0.27 | -1.37 | -1.15 |
| EC0177 | 5.07 | 3.94 | 3.94 | 3.94 | 3.94 | 3.94 | -2.46 | 2.60 | **1.01** | **1.33** | -5.07 | -5.07 | **-4.42** | -1.77 | 4.42 | 2.91 | -5.07 | -4.42 | -4.42 |
| EC0178 | 5.15 | 3.85 | 3.85 | 3.85 | 0.82 | 3.85 | -2.55 | 0.53 | **-4.50** | -1.56 | -5.15 | -5.15 | -5.15 | -2.62 | **4.27** | -0.91 | -5.15 | -5.15 | -4.50 |
| EC0334 | 5.22 | 3.78 | 3.78 | 3.78 | 3.84 | 3.83 | 0.18 | -1.29 | -0.09 | **3.74** | -4.57 | -5.22 | -5.22 | -5.22 | -1.36 | -1.85 | -1.85 | -1.33 | -1.47 |
| EC1170 | 5.39 | 3.61 | 3.61 | 3.61 | 3.61 | 3.61 | 3.61 | 3.61 | 3.61 | 3.61 | 3.61 | 3.61 | 3.61 | -0.98 | 3.61 | 3.61 | -1.09 | -0.78 | -0.76 |
| EC0172 | 5.19 | 3.81 | 3.81 | 2.81 | 3.81 | 3.81 | -5.19 | -1.39 | **-5.19** | **-4.39** | **-5.19** | **-5.19** | **-5.19** | **-5.19** | **4.02** | **3.41** | -1.24 | -1.31 | -1.41 |
| EC0299 | 5.18 | 3.82 | 3.82 | 3.82 | 4.08 | 4.19 | -1.42 | 3.82 | -5.18 | -5.18 | -5.18 | -5.18 | -5.18 | -5.18 | 3.82 | -1.12 | 2.09 | 3.82 | 3.82 |
| EC0360 | 5.22 | 3.78 | 3.78 | 3.78 | 3.77 | 3.79 | 0.48 | 1.26 | 0.51 | -5.22 | -5.22 | -5.22 | -5.22 | -4.33 | **3.78** | **3.78** | -0.48 | 0.42 | **3.78** |
| EC0564 | 5.46 | 3.54 | 3.54 | 3.54 | 3.54 | 3.54 | 3.54 | -2.42 | 3.54 | 2.54 | 3.54 | 3.54 | 3.54 | -2.72 | **0.54** | **1.54** | **2.18** | **2.20** | **2.54** |
| EC0567 | 5.20 | 4.35 | 4.24 | 4.28 | 4.10 | 4.26 | 3.93 | -1.32 | 3.80 | 3.80 | 2.80 | 3.80 | 3.80 | -1.39 | **3.80** | **3.80** | 0.37 | **1.99** | **1.15** |
| EC0391 | 5.34 | 3.66 | 3.66 | 3.66 | 2.66 | 3.66 | 3.66 | 3.66 | 3.66 | 3.66 | 3.66 | 3.66 | 3.66 | -2.02 | 3.66 | 0.74 | -1.51 | 1.84 | 3.66 |
| EC1488 | 5.20 | 3.80 | 3.80 | 3.80 | 3.80 | 3.80 | -0.14 | -1.53 | -0.11 | -2.71 | -3.51 | **3.80** | 0.80 | -0.18 | -0.47 | 3.80 | -0.39 | -1.36 | -1.19 |
| EC1522 | 5.23 | 3.77 | 3.77 | 3.77 | 2.77 | 3.77 | -0.49 | 0.80 | -0.94 | -3.83 | 0.57 | -5.23 | -5.23 | -2.53 | -0.51 | -1.86 | 1.08 | 0.20 | -0.82 |
| EC1645 | 5.20 | 3.80 | 3.80 | 3.80 | 2.80 | 3.80 | -0.26 | -0.17 | -0.75 | -0.22 | -4.80 | -5.20 | -1.45 | -3.91 | -0.71 | 0.10 | -1.60 | 0.80 | -0.59 |
| EC1655 | 5.16 | 3.84 | 3.84 | 3.84 | 3.84 | 3.84 | -0.02 | -0.12 | -0.43 | -4.08 | -2.20 | -2.58 | 0.17 | -5.16 | 0.19 | 0.30 | -1.32 | 1.02 | -1.31 |
| EC1678 | 5.13 | 3.87 | 3.87 | 3.87 | 2.87 | 3.87 | -0.33 | 3.87 | -0.87 | **3.87** | -3.03 | -3.87 | -5.13 | -3.00 | -0.93 | -0.96 | 3.87 | 0.48 | -0.62 |
| EC1729 | 5.22 | 3.78 | 3.78 | 3.78 | 3.78 | 3.78 | 1.14 | -1.32 | 0.38 | 0.55 | 0.84 | -0.76 | -1.95 | **-3.29** | **0.68** | -1.11 | -0.25 | -1.24 | -0.95 |
| EC1792 | 5.36 | 3.64 | 3.64 | 3.64 | 3.64 | 3.64 | -0.63 | -0.96 | -3.10 | -2.59 | -3.67 | -5.36 | -5.36 | -4.55 | -0.75 | -0.93 | -0.45 | -0.86 | -0.92 |
| EC0462 | 5.12 | 3.88 | 3.88 | 2.88 | 4.03 | 3.96 | -1.41 | 0.27 | -1.34 | 0.18 | 0.24 | -2.91 | -0.81 | -2.36 | -2.06 | -2.49 | -2.00 | -2.08 | -2.90 |
| Bactericidal activity (≥3log10 CFU/mL decrease from the initial inoculum) is highlighted in grey; Bactericidal combinations which are synergistic (≥2log CFU/mL decrease compared to the most active single antibiotic) is indicated in bold typeface; Antagonism (≥2log CFU/mL increase compared to most active single antibiotic) is indicated in red font.  ATM, aztreonam; DOR, doripenem; FEP, cefepime; MEM, meropenem; PMB, polymyxin B; TAZ, piperacillin-tazobactam; TGC, tigecycline | | | | | | | | | | | | | | | | | | | |

**
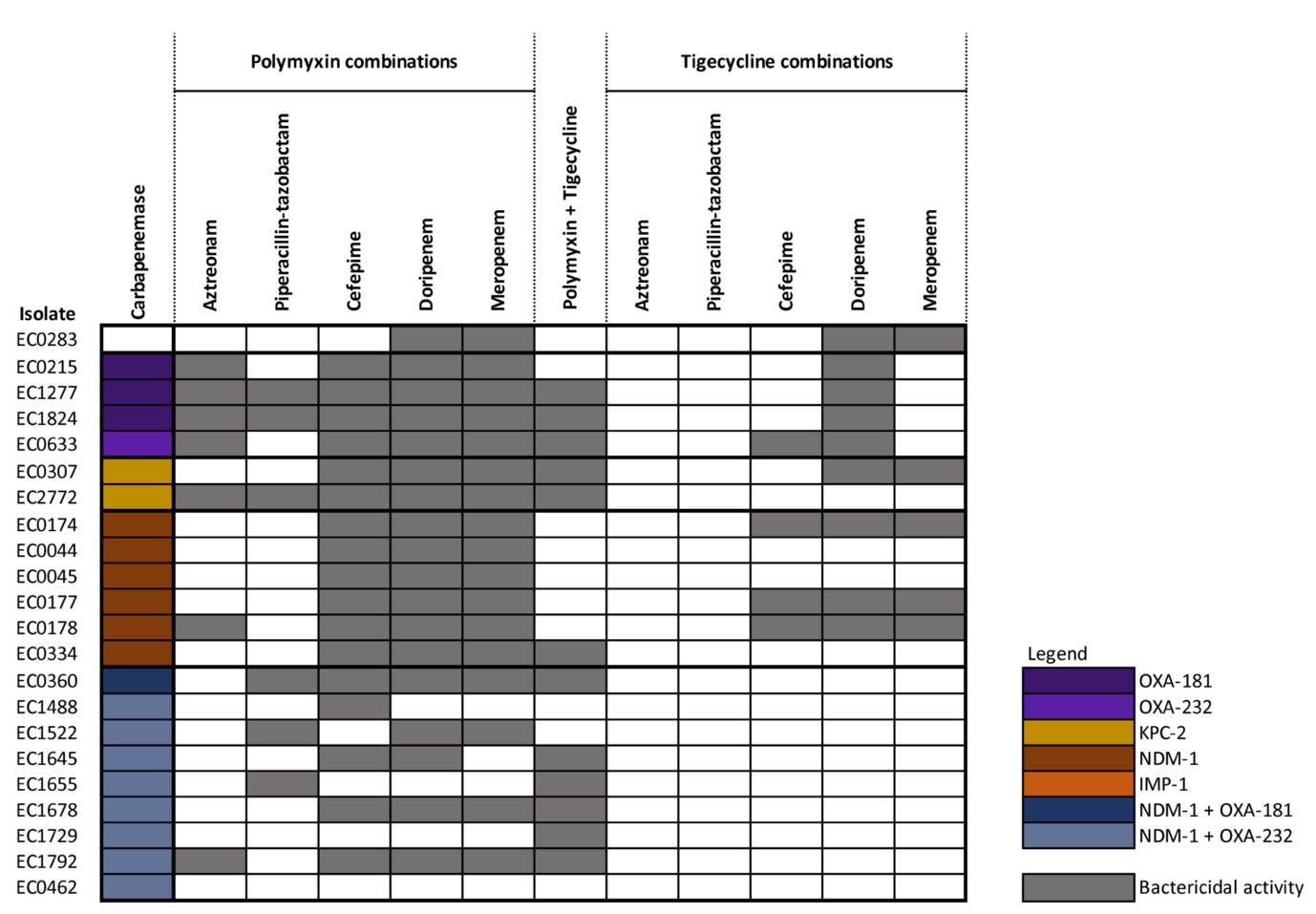
**

**Figure S1.** Bactericidal activities of the various antibiotic regimens against 22 polymyxin and tigecycline-susceptible CRKP where monotherapies did not demonstrate bactericidal activity.

**References**

1. LaPlante KL, Sakoulas G. 2009. Evaluating aztreonam and ceftazidime pharmacodynamics with Escherichia coli in combination with daptomycin, linezolid, or vancomycin in an in vitro pharmacodynamic model. Antimicrob Agents Chemother 53:4549-55.

2. Tam VH, McKinnon PS, Akins RL, Drusano GL, Rybak MJ. 2003. Pharmacokinetics and pharmacodynamics of cefepime in patients with various degrees of renal function. Antimicrob Agents Chemother 47:1853-61.

3. Shea KM, Cheatham SC, Wack MF, Smith DW, Sowinski KM, Kays MB. 2009. Steady-state pharmacokinetics and pharmacodynamics of piperacillin/tazobactam administered by prolonged infusion in hospitalised patients. Int J Antimicrob Agents 34:429-33.

4. Jaruratanasirikul S, Wongpoowarak W, Kositpantawong N, Aeinlang N, Jullangkoon M. 2012. Pharmacodynamics of doripenem in critically ill patients with ventilator-associated Gram-negative bacilli pneumonia. Int J Antimicrob Agents 40:434-9.

5. Tam VH, Schilling AN, Nikolaou M. 2005. Modelling time-kill studies to discern the pharmacodynamics of meropenem. J Antimicrob Chemother 55:699-706.

6. Sandri AM, Landersdorfer CB, Jacob J, Boniatti MM, Dalarosa MG, Falci DR, Behle TF, Bordinhao RC, Wang J, Forrest A, Nation RL, Li J, Zavascki AP. 2013. Population pharmacokinetics of intravenous polymyxin B in critically ill patients: implications for selection of dosage regimens. Clin Infect Dis 57:524-31.

7. Rodvold KA, Gotfried MH, Cwik M, Korth-Bradley JM, Dukart G, Ellis-Grosse EJ. 2006. Serum, tissue and body fluid concentrations of tigecycline after a single 100 mg dose. J Antimicrob Chemother 58:1221-9.
